# Application of Quercus salicina extract in the management of urolithiasis

**DOI:** 10.3389/fmed.2022.960292

**Published:** 2022-09-20

**Authors:** Pei Lu, Junyan Pu, Yiping Zong, Zijie Wang, Peng Han, Min Gu

**Affiliations:** ^1^Department of Urology, The First Affiliated Hospital of Nanjing Medical University, Nanjing, China; ^2^Department of Urology, The Affiliated Yixing Hospital of Jiangsu University, Yixing, China

**Keywords:** Quercus salicina extract, urolithiasis, anti-oxidative, stone formation, clinical application

## Abstract

Since 1969, an herbal medicine extracted from Quercus salicina Blume/Quercus stenophylla Makino (QS) has been clinically used for the management of urolithiasis in Japan. Historically, the decoction of leaves and shoots of QS trees was popularly utilized as a folk prescription to remove urinary calculi. This study was designed to perform a brief review of the updated progress of QS extract for urinary stones based on previous studies. A comprehensive literature search was conducted in multiple electronic databases, including Web of Science, PubMed, and EMBASE, and relevant data on QS extract were extracted. As a result, the major mechanism of QS extract for urolithiasis is observed to be closely related to the anti-oxidative activities according to recent studies, leading to inhibition of the accumulation of renal calcium and prevention of stone formation and recurrence of stones. As for the effect of discharging stones, loosening the upper urinary tract has also been noticed recently. More extensive studies are still necessary to systemically evaluate the individual dosage, drug safety, and targeted stone types.

## Introduction

Urolithiasis is one of the common and detrimental diseases, affecting about 10–15% of the population around the world (1). In Asia, the prevalence is about 1–19.1%, varying with different social conditions and geographical positions. While the prevalence of urolithiasis keeps increasing these years, the recurrence rate reaches up to 21–53% after 3–5 years, and the lifetime risk of recurrence rate is about 60–80% (2). As a result, the urinary stone disease is a great burden for the health care system, which reminds us to pay attention to such disease (3).

According to the latest guidelines (4–7), current urinary stone management was generally classified into two types of treatments: surgical treatment and conservative treatment. For stones larger than 10 mm in diameter, surgical treatment is recommended according to international guidelines, such as percutaneous nephrolithotomy (PCNL), retrograde intrarenal surgery (RIRS), extracorporeal shockwave lithotripsy (ESWL), and ureteroscopic lithotripsy. The final selection of these operative methods is mostly determined by the positions and sizes of the stones. Aside from these operative treatments, conservative treatment is recommended for the spontaneous expulsion of ureteral stones, especially those smaller than 10 mm in diameter. Conservative treatment includes medical expulsion therapy (MET) and periodic follow-up, conventional oral drugs utilized in MET are alpha-blockers, calcium channel blockers, and PDE5 inhibitors, which can help relax ureteral passage and discharge stones.

In the 1960s, the extract of Quercus Salicina entered clinical application in Japan as a new oral administration for urolithiasis. Some controlled studies conducted in Japan proved that QS extract could exert its biological function by discharging upper urinary tract calculi (8, 9), while a series of randomized controlled clinical studies were carried out later to evaluate the efficacy and safety of the QS extract in the management of patients with kidney ureteral calculi, indicating that QS extract could significantly improve stone discharge rate, shorten the calculi discharge time and relieve back pain symptom (10–14). In cases of patients with larger kidney stones (larger than 10 mm in diameter), QS extract was also utilized after operative treatment, such as ureteroscopic lithotripsy, to increase the lithagogue rate (15, 16). Subsequently, a few studies involved the specific mechanism about the function of curing and preventing urolithiasis of QS extract were performed, indicating that the medical properties of QS are mostly due to its antioxidative capacity (17, 18).

To date, the administration of QS extract was limited in East Asian countries, while the biological function and its molecular mechanisms remain greatly lacking extensive research. Furthermore, various clinical issues were noticed in clinical use, hindering its wide application. Thus, this review focuses exclusively on the current progress of applications of QS based on its biological functions, especially on the clinical use for urolithiasis and related mechanisms.

## Materials and methods

A comprehensive literature search was conducted in multiple electronic databases, including Web of Science, PubMed, and EMBASE, and relevant data on QS extract were extracted.

### Quercus salicina Blume extracts

Quercus trees are oak species native to Japan and Korea. Aside from the resource for food and construction, researchers analyzed the components of Quercus species and found out that, especially, Quercus salicina Blume, were rich in phenolic compounds (19). Through the extraction of QS, 5 main components were isolated, named: (1) D-threo-guaiacylglycerol 8-O-β-D-(6'-O-galloyl)glucopyranoside, (2) 9-methoxy-D-threo-guaiacylglycerol8-O-β-D-(6'-O-galloyl)glu copyranoside, (3) 6"-O-galloyl salidroside, (4) methyl gallate, and (5) quercetin (20). The first four components are all phenolic compounds, generally consisting of phenolic acids and flavonoids, which have the property of antioxidation. Antioxidants are indispensable to defend against the damage of oxidative stress, caused by free radicals and reactive oxygen species (ROS), which can be classified into two categories: preventive antioxidants, including superoxide dismutase (SOD), catalase (CAT), glutathione peroxidase (GTx), and radical scavenging antioxidants, including vitamin C, and flavonoid (21). As mentioned above, QS extract comprises large amounts of flavonoids, which belong to radical scavenging antioxidants, so QS extract prevents the injury caused by free radicals.

At present, QS extract has been mainly used in the fields of medicine, cosmetics, and food, the applications are listed in Table 1 (23–28). Basically, the majority of biological functions are related to antioxidation.

**Table 1:** Major medical functions and applications of QS extract.

**Application**	**Major functions**	**References**
Urolithiasis	Protect the renal tubular cells from the injury caused by free radicals and prevent stone formation	(18)
Rheumatoid arthritis	Suppress oxidative stress in rheumatoid arthritis	(22)
Anti-diabetic	Provide cytoprotective effect against oxidative stress and increase the insulin secretion of pancreatic b cells	(23)
Cardiovascular disease	Endothelium-dependent relaxation of coronary artery *via* activation of endothelial NO synthase	(24)
Allergic diseases	Anti-allergic and atopic effects	(25)

### Clinical applications of QS extracts in the treatment of urolithiasis

Nowadays, QS extract has been applied to clinical treatment alone or combined with operative management as oral administration. We have summarized studies conducted to testify to the therapeutic efficacy of urinary stones, and the results were systematically shown in Table 2.

**Table 2:** Results of major research concerning the efficacy of urocalun for urinary stones.

**Year**	**Study design**	**Patients**	**Conclusions**
1967 (8)	Clinical study	21 patients with ureteral stones, 11 patients with renal stones, all administered QS extract capsule	QS extract capsule was effective to discharge stones in 75% of the cases with ureteral stones, but no changes happened to incarcerated calyceal stones
1969 (29)	Double-blind controlled clinical study	106 cases with upper urinary stones, half in placebo group and half in QS group	the stone discharge rate and stone down rate in QS group were higher than that in placebo group, and the differences were statistically significant
2002 (10)	Open-label clinical trial	60 patients with ureteral calculi < 1 cm received QS extract capsule orally	75% cases eliminated the calculi, mostly in upper and lower ureter, no stone discharge in the middle ureter
2009 (18)	Animal experiment	rat calcium oxalate urolithiasis, administered with QS extract	QS extract restrained the renal calcium accumulation induced by oxidative damage in kidneys
2011 (11)	Open-label clinical trial	75 patients with ureteral calculi < 1 cm received QS extract capsule orally	74% cases discharged the stones, 20% cases showed stone descending, mostly in lower ureter
2015 (12)	Prospective multicenter randomized controlled clinical study	88 cases of ureteral stones, 23 cases of renal stones in QS group and 84 cases of ureteral stones, 26 cases of renal stones in control group	clinical symptoms were relieved greatly and the cure rate was higher in QS group, the QS extract capsule was safe and effective in the treatment for stones < 1 cm
2015 (30)	Animal experiment	60 mice divided into 4 groups: control group, renal calcium oxalate group, low dose QS group, high dose QS group	the concentration of Ca^2+^ and oxalate in urine in QS groups were decreased, and the activity of SOD and GSH-Px increased, such changes were more apparent in high dose QS group, indicated that QS extract increased the stone-preventing effect and antioxidant activity
2016 (13)	Randomized controlled clinical study	286 patients with urinary calculi < 1 cm divided into 2 groups: control group and QS group	in 4-week-course, the stone remove rate was significantly higher in QS group, especially in week 1 and week 2, proving significant clinical efficacy and safety in treating small urinary calculi
2019 (16)	Retrospective controlled clinical study	62 cases with residual stones after USL divided into 2 groups: ESWL and ESWL with QS extract capsule	ESWL with QS extract capsule brought higher stone discharge rate and lower retreatment rate
2019 (15)	Retrospective controlled clinical study	50 cases after USL for renal stones 2–3 cm divided into QS group and control group	the stone free rate was much higher in week 2, week 3, week 4, and the stone discharge time was shorter in QS group, suggested QS extract capsule increased the stone discharge rate after USL
2021 (31)	Animal experiment	rat model with renal calcium oxalate stone, intervened with QS extract capsule for 4 weeks	QS extract capsule inhibited the formation of renal calcium oxalate stones, attenuating the oxidative stress process and protecting renal tubular cells by reducing autophagy and apoptosis through MPAK signaling pathway
2021 (14)	Randomized controlled clinical study	148 patients with intramural ureteral stones < 1 cm divided into 3 groups: tamsulosin, tamsulosin + Potassiun sodium hydrogen citrate, tamsulosin + QS extract capsule	QS extract capsule + tamsulosin performed better efficacy for stones < 1 cm in intramural ureter than other two groups, the combination therapy was considered to be more helpful than single classical drugs

Patients with kidney ureteral calculi that are less than 10 mm in diameter are considered to be able to pass stones by medication, while one prospective multicenter randomized controlled clinical study evaluated the efficacy of QS extract by providing a 4-week-treatment for these patients (12). A total of 111 patients in the experiment group administered QS extract capsules, 2 pills every time, 3 times a day, and 110 patients in the control group administered lithagogue powder, 1 bag every time, 3 times a day. After a 4-week-treatment, some clinical symptoms of patients in the experiment group, like low back pain or/and painful urination, and urinary frequency, were greatly relieved, and evaluating indexes like stone discharge rate, stone down rate, and stone expelling failure rate were 56.8, 12.6, 30.6, 39.1, 10, and 50.9% in experiment group and control group, respectively. The patients' conditions greatly improved in the experimental group, the total effective rate was 69.4%, much higher than in the control group, which was 49.1%, proving the overall efficiency of the QS extract capsule. In another study, the stone removal rate increased from 27 to 74% as the medication time was prolonged, and the stone removal rate was also variant on account of different positions in the urinary tract based on results of the plain film of kidney-ureter-bladder; it seemed to be most helpful for lower ureteral calculi (11), but it is still to be testified as the cases contained in this study were not enough.

Apart from monotherapy, combined treatments, including QS extract capsules and other conventional drugs or QS extract capsules administered after surgical management, were also reported. Conventional drugs, like a1-blockers, are recommended for urinary stones (< 10 mm) expulsion as they decrease the ureteral peristaltic frequency, smooth muscle tonus, contractile force, and increase ureteral flow. In a recent Chinese study, the author compared the effectiveness in 3 groups: the observation group used tamsulosin (a1-blocker) alone, the control group used tamsulosin and Potassium Sodium Hydrogen Citrate Granules, and the experiment group used tamsulosin and QS extract capsule. The stone discharge rates in the observation group, control group, and experiment group were 36, 78, and 82%, indicating that the combined treatment, tamsulosin and QS extract capsule, was the most effective (14). The clinical efficacy of QS extract was also confirmed in patients with kidney stones (2 to 3 cm) after receiving ureteroscopic lithotripsy, the effect of expediting stone discharge was better in the QS extract capsule group than that in the lithagogue powder group (15). Oral administration of QS extract capsule combined with ESWL (extracorporeal shock-wave lithotripsy) was also reported as a treatment for residual stones after ureteroscopic lithotripsy, the study concluded that such combination therapy was much more efficient than ESWL alone, not only increasing the stone-free rate but also decreasing the retreatment rate (16).

As we know, calcium oxalate has constituted the majority of urinary stones in recent years (32) and previous studies showed that the urinary oxalate levels of patients with urolithiasis were higher, meanwhile, increasing lipid peroxides and urinary tubular enzymes were found, indicating that the exposure to oxalate could result in lipid peroxidation and tubular cells injuries, primarily the proximal tubular cells (33). Such changes in the oxidant-antioxidant balance might be due to the toxic substance brought by the exposure to oxalate initially, and in the late stage, leukocytes infiltrated and antioxidant enzyme activities decreased, causing the kidney to remain under excessive oxidative stress (34). Free radical production elicited by peroxidation was related to the concentration of oxalate, but some researchers proposed that even though there was a tiny increase in urinary oxalate levels, it might still contribute to the progression of renal diseases (35). Later studies demonstrated that oxalate-induced free radical production in renal tubular epithelial cells was through the activation of NAD(P)H oxidase *via* cytokine TGF-β1 induction, and free radical scavengers could make this peroxide production decrease noticeably (36). These researches provide us the evidence that antioxidant therapy can prevent oxalate-mediated peroxidation and cell injuries caused by peroxidation; therefore, there would be less cellular debris for calcium oxalate crystal nucleation and aggregation, suspending the kidney stone formation and preventing stone recurrence. That is how QS extract works as lithophytic agent. Animal experiments have provided evidence that QS extract can inhibit the formation of renal calcium oxalate stone and the oxidative process. The researchers established the model mice with renal calcium oxalate stones by Glycol, and the administration of QS extract could decrease the concentration of Ca^2+^ and oxalate in the urine, and increase the activity of SOD and GSH-Px (30). Further mechanism illustrated that QS extract capsule inhibited the oxidative process induced by calcium oxalate crystallization and reduced the phosphorylation of MPAK signaling pathway, then the process of autophagy and apoptosis through MPAK signaling pathway was attenuated (31), which could protect the renal tubular epithelial cells and prevent the calcium oxalate crystal adhesion.

### Safety

Mild side events, including nausea and vomiting, occurred in several cases, whereas there were no significant differences in incidence between the intervention group and control group in all these studies; after symptomatic treatment, these reactions were eased, and there were no indications of drug withdrawal. No severe side effects happened in these clinical trials yet. Furthermore, researchers did not exclude the possibility that such reactions might be related to nephrocolic instead of the medication. It remains to be investigated whether there are specific side effects of QS extract.

Japanese researchers also made a study of clinical equivalence of two different formulations of QS extract, capsule, and tablet, to examine whether these formulations would have an impact on the medicine potency. Results pointed out that both formulations exerted the same clinical effectiveness, while the miniaturized tablet might be better for medication compliance since it is more convenient for swallowing (37).

## Limitations

As mentioned above, a 4-week treatment of QS extract capsule was usually contained, but no studies adopted a shorter or longer treatment period, although the stone discharge rate of patients administering QS extract capsule was higher, there was no significant difference in week 3 and week 4 (13). Therefore, the duration of QS extract capsule treatment needs to be further investigated whether a shorter course is efficient enough or a longer course is required to ensure the clearance of stones. Second, the dosage of the QS extract capsule in these studies was 450 mg taken 3 times per day. It is still unknown if there is a cytotoxic effect of QS extract capsule in clinical dosage and whether it would cause a medicament liver lesion or other damage when a longer course is required.

Importantly, no drug use report for children, pregnant, or lactating women was reported up to now. Moreover, the QS extract capsule performed different strengths on discharging stones in different positions of the urinary tract, which requires more investigation to clarify the concrete differences such medicine could bring about. Except for calcium oxalate calculus, urinary calculi include cystine calculus, magnesium ammonium phosphate calculus, and calcium phosphate calculus; the effect of the QS extract capsule on these stones remains to be studied to provide a clinical guide as an individualized treatment for patients with different types of stones.

As to the mechanism of QS extract capsule on the formation of urinary calculi, the protection for renal tubular epithelial cells through antioxidant activity against oxidative stress damage is considered as the main factor, inhibiting the crystals accumulation and stone formation. The researchers also found that acute renal tubular injury was relieved, and if intervened with a QS extract capsule in rat model with renal calcium oxalate stones (31), the concentration of Cr, BUN was decreased (30), suggesting that once the QS extract capsule could improve the renal function, there might be other ways for QS extract capsule to influence the renal tubular epithelial cells beside from antioxidation, and the corrected mechanism is under discussion, which could provide a new insight for the prevention and measures of urolithiasis.

## Conclusions

QS extract has been utilized for the management of urolithiasis for several years, while most of the effects were attributed by its medical properties to the antioxidative activities. Clinical studies and animal experiments have proven that QS extract is of high efficacy and safety in the treatment of urinary calculi when used alone or in combination therapy. Taken together, QS extract improves the stone-free rate and resubmission rate in urolithiasis. With the advance in medical technology, the treatment for urolithiasis has improved dramatically, but its prevention still lacks effective medicine. The role of renal tubular cells' protection of QS extract by antioxidation may paint a fascinating and incomplete picture to take measures in preventing the calcium oxalate stone formation and recurrence.

## Author contributions

PL: substantial contributions to the conception and design of the work, interpretation of data for the work, and manuscript revision. JP: the acquisition, analysis, and interpretation of data for the work and drafting the work. YZ: substantial contributions to the conception and design of the work and manuscript revision. ZW: manuscript revision and agreement to be accountable for all aspects of the work in ensuring that questions related to the accuracy. PH: agreement to be accountable for all aspects of the work in ensuring that questions related to the accuracy and integrity of any part of the work are appropriately investigated and resolved. MG: substantial contributions to the conception and final approval of the version to be published. All authors contributed to the article and approved the submitted version.

## Funding

This work was supported by the National Natural Science Foundation of China [Grant Nos. 82170769 and 81870512], Project of Jiangsu Province for Important Medical Talent [Grant No. ZDRCA2016025], the 333 High-Level Talents Project in Jiangsu Province [Grant No. BRA2016514], and the Standardized Diagnosis and Treatment Research Program of Key Diseases in Jiangsu Province [Grant No. BE2016791].

## Conflict of interest

The authors declare that the research was conducted in the absence of any commercial or financial relationships that could be construed as a potential conflict of interest.

## Publisher's note

All claims expressed in this article are solely those of the authors and do not necessarily represent those of their affiliated organizations, or those of the publisher, the editors and the reviewers. Any product that may be evaluated in this article, or claim that may be made by its manufacturer, is not guaranteed or endorsed by the publisher.
